# Patient’s pre-operative dental anxiety is related to diastolic blood pressure and the need for post-surgical analgesia

**DOI:** 10.1038/s41598-020-66068-9

**Published:** 2020-06-08

**Authors:** Javier Fernandez-Aguilar, Isabel Guillén, María T. Sanz, Mar Jovani-Sancho

**Affiliations:** 1 Dentistry Department, Faculty of Health Sciences, Universidad Cardenal Herrera-CEU, 46115 Alfara del Patriarca, Valencia, Spain; 2 Pharmacy Department, Faculty of Health Sciences, Universidad Cardenal Herrera-CEU, 46115 Alfara del Patriarca, Valencia, Spain; 3 Didactics of Mathematics Department, University of Valencia, Valencia, 46022 Spain

**Keywords:** Psychology, Medical research

## Abstract

In order to study the relationship of the patient’s anxiety level from Corah’s Dental Anxiety Scale (DAS) vs different physiological parameters: pre and post-operative blood pressure, and pre and post-operative heart rates, and subsequently, relate the results to the patient’s post-operative anti-inflammatory analgesic need, 185 patients requiring a simple dental extraction were recruited. They filled out the DAS in the waiting room prior to their procedure and once in the examination room, their preoperative blood pressure and heart rate was measured. Once the dental extraction had been completed, their blood pressure and heart rate were measured again. Before leaving the clinic, the patient was given an analgesic form in which they had to indicate whether or not they had required analgesia after the procedure. Diastolic blood pressure (DBP) showed statistically significant differences between pre-operative and post-operative (*P* = 0.001). DAS was related with pre-operative diastolic blood pressure (pre-DBP) (*P* = 0.001) and post-operative diastolic blood pressure (post-DBP) as well as pre-operative heart rate (pre-HR) (*P* = 0.027) and post-operative heart rate (post-HR) (*P* = 0.013). Patients with high levels of DAS tend to take more Ibuprofen 400 mg (*P* = 0.038). The different levels of anxiety will determine what type of anti-inflammatory analgesia the patient will take, if necessary.

## Introduction

The fear and anxiety felt by the patients seen by odontologists is a daily reality in dentistry. Approximately, one out of every seven patients treated in western countries feels high levels of anxiety and/or fear^1^. This occurs mainly with those patients who believe their visit to the dentist will be a painful experience^2^ or who have previously had painful experiences in the dental clinic^3^.

The patient may experience different negative reactions when facing dental treatment, such as *anxiety*, in which an imagined future threat is perceived, which leads to an increased activation of the sympathetic nervous system (for example, sitting in the waiting room of the dental clinic before a procedure); *fear*, as a response to a real action which is perceived as imminent (in the moment when a patient is sitting in the dentist’s chair just before undergoing anaesthesia or a procedure)^4^; or even *dental phobia*, which is included within phobias related to “blood-injections-fear” and defined in the classification of “Diagnostic and Statistical Manual of Mental Disorders” (DSM IV) as a pronounced, persistent, excessive and irrational fear caused by the presence of a specific object or situation^5^. It is of interest to highlight that the same publication indicates that dental phobias are accompanied by an intense vasovagal response. However, not all treatments cause anxiety or fear in the patient. According to a study by Oosterink *et al*. published in 2009, in the 67 odontological situations analysed, those that stand out are, in first place, fear of oral surgery and, in fifth place, fear of dental extraction^3^.

Many different scales have been used for a long time to evaluate a patient’s level of anxiety before treatment. Corah’s Dental Anxiety Scale (DAS), formulated by Norman Corah, has been the most widely used since its publication in 1969^6^. The DAS consists of four questions for the patient with five possible answers. The numerical value obtained is used to classify the patient according to his/her level of anxiety. Later, in the year 1995, the DAS was modified by Humphris *et al*., establishing the Modified Dental Anxiety Scale (MDAS). This test includes an additional question regarding the anxiety felt by the patient in regards to a local anaesthetic injection^7^. It is important to take into account that anxiety or fear regarding dentistry and dental treatment does not only result from personal experiences but also, indirectly, from the experiences of family and friends^8^. The development of this emotional state can result in delaying appointments set up by the dentist or even missing these. In 1984, Berggren and Meynert proposed that, as a consequence, a vicious cycle is produced: when a patient avoids getting a dental pathology treated, oral health and quality of life worsen, resulting in a larger number of necessary dental treatments^9^. A patient who has developed a medium-high level of anxiety will try to avoid regular visits, only going when absolutely necessary^10^. This translates to the patient’s oral condition getting worse in general and only visiting the clinic once the pathology is at an advanced state^11^ as demonstrated in the study carried out recently by Levin *et al*., in which advanced stages of aggressive periodontitis are connected to varying levels of anxiety using the DAS^12^.

As previously stated, fear and pain can also be determined by previous experiences of the same patient. As demonstrated in a study in 1980, the higher the level of fear, the patient will remember the treatment as more painful than patients with low levels of fear^13^. This would reinforce the previous idea that this type of patient will defer treatment over time, fundamentally reaching more interventional treatments. However, fear and anxiety do not only appear in surgical treatments. In conservative treatments such as root canal treatment, patients are also found to have higher expectations of pain than they actually feel afterwards during treatment^14^.

Furthermore, it has been observed that when the patient is already in the dental clinic or in the dental chair waiting to be treated, different physiological parameters vary depending on the circumstances of each patient, in adults as well as children^15,16^. In each group, heart rate (HR) and blood pressure (BP) vary depending on the treatment that the patient is undergoing^16^. Therefore, this leads us to believe that it is not only a question of clinical treatment, but that we should also consider that we have a patient in our hands who normally feels upset or scared, and thus, the variables of fear and anxiety should be calibrated and taken into account as part of the dental treatment. In regards to the most commonly used local anaesthetics in dentistry (articaine and lidocaine), both are classified as “medium” in character due to their anaesthetic properties, and, despite having a similar latency time, articaine lasts longer after its administration. Both have been used with vasoconstrictors in the study (epinephrine 1:100.000) in order to evaluate their possible relation in the influence of the physiological parameters studied^17–19^.

In this study different physiological variables have been analysed both in the pre and post-operative periods in patients about to undergo a dental extraction. Later, it was analysed whether the physiological response observed corresponded to the values obtained in the DAS, and lastly, how all this influences post-operative analgesia in regards to type of medicine and analgesic time needed. Therefore, our working hypothesis is to assess whether patients with more anxiety will tend to take more analgesic or anti-inflammatory medication.

## Patients and methods

### Information regarding the study population

The sample population consisted of 185 Caucasian patients, 92 men and 93 women, with an average age of 56 ± 18.1. The patients were divided into two age groups: Group A was from 18 to 45 years old (n = 57, 30.8%) and Group B was from 46 to 90 years old (n = 128, 69.2%). The division was made to analyse if there were changes in the variables studied with respect to age. The study was carried out between June 2017 and October 2018 in a dental clinic located in the town of Albal (Valencia). The patients underwent a simple dental extraction after an exhaustive diagnosis and a periapical radiograph which justified not restoring the tooth or the patient deciding against restoring it. The study was approved by the corresponding Ethics Committee in the CEU-Cardenal Herrera University in Valencia (CEI17/073). All treatments were performed in accordance with relevant guidelines and regulations. Each patient had to voluntarily sign an informed consent form in order to take part in the study.

### Inclusion and exclusion criteria

The inclusion criteria in this study were: (1) patients of legal age; (2) healthy patients and/or patients whose systemic pathology is controlled by a medical specialist and can be treated safely by a dentist; (3) patients who had to undergo a simple dental extraction and who had never been treated by the dental operator; (4) patients who were able to understand the objective of the study and participate correctly.

On the other hand, any patients who, for any reason, did not comply with the criteria detailed above were excluded, such as: (1) patients suffering from (or suspected of suffering of) a systemic illness without adequate control under a specialist, or patients who could not be treated by the dentist until having received adequate medical control due to their medical condition or situation; (2) patients who had to undergo dental extraction previously diagnosed as surgical and; (3) patients who for any reason had to wait more than thirty minutes in the waiting room after the scheduled time for their extraction (in order to normalise stress due to waiting time).

### Procedure

Once in the waiting room with no other patients, before being seen by the dentist, each patient filled out Corah’s Anxiety Test (DAS), a test which is specially designed to measure a patient’s fear and anxiety when going to the dental clinic. The test consists of four multiple choice questions in which the patient may only select one answer. From this a series of values is obtained: 4–8 (no anxiety), 9–12 (moderate anxiety), 13–14 (high anxiety), and 15–16 (severe anxiety or phobia). All the extractions were made by a single operator. Patients did not know their dentist, the dental extraction was the first treatment with him, and the dentist did not know the value of DAS until the tooth extraction was finished.

After entering the examination room and before being anaesthetised, the patient’s blood pressure and heart rate was measured using the validated “Omron M2 Compact” blood pressure monitor (Omron Healthcare Co. Kyoto-Japan). When gathering data regarding the patient and the tooth being extracted, different variables were taken into consideration, such as: (a) the sex and age of the patient, (b) the condition of the pulp of the tooth to be extracted (whether the tooth had root canal treatment or not), (c) the type of anaesthesia used (articaine or lidocaine) in order to know if the type of anaesthesia affects the physiological variables, (d) periodontal state of the tooth (level of tooth mobility classified according to: level I, level II or level III), (e) prior intake (or not) of antibiotics due to an infection which would justify the use of antibiotics, and (f) total extraction time in seconds (a stopwatch was started when the extraction began and stopped when it successfully ended, with no pauses if the extraction had to be interrupted, even due to fear or anxiety)

Half of the sample population was anaesthetised with articaine 4% (n = 46 men and n = 46 women) and the other half with lidocaine 2% (n = 46 men and n = 47 women) both with epinephrine as vasoconstrictor. The assignment of the anaesthetic agent was random, the first half of the sample was anaesthetized with articaine 4% and the second half with Lidocaine 2%. After checking the anaesthetic was correct, the extraction was initiated. Once the extraction was finished, the patient’s blood pressure and heart rate were again measured. Both registers of blood pressure and heart rate (pre-operative and post-operative) were taken with the patients sitting in the chair in upright position. Pre-operative register was measured when the patient arrived at the dental surgery and post-operative register was measured immediately after the extraction.

The patients were given a form to fill in to evaluate analgesia; this was in the form of a test in which they had to answer whether they had suffered from post-operative pain, and in the case that they had, they had to register the number of days the pain had lasted (from the same day of the extraction until up to a week later) and which analgesic drug had been used to calm the pain (the following options were provided: paracetamol 650 mg, ibuprofen 400 mg or “other” in case of allergy or incompatibility with either of these). The selection of these two agents was done because they are the most widely used to eliminate the usual pain in patients and in both cases no prescription is required for their use. The doses were due to the attempt to achieve maximum benefit with the minimum dose of medication. All patients included in the study had to return the form when they came back for an appointment arranged for a week after the extraction.

### Statistical analysis

The data collected was treated with the *IBM SPSS Statistics 20.0* programme *(IBM, Armonk, NY, USA)*, which was used to carry out inferential, descriptive and statistical studies.

In the inferential analysis, different tests were used depending on the nature of the variables being studied. The existence of some type of association between two or more variables represents the presence of some kind of tendency or pattern which paired together the different values of these variables. At a higher significance level of *P*-value < 0.05 the null hypothesis will be rejected in each of the aforementioned tests.

## Results

### Population study

The 18-45 age group had a total of 57 patients (30.8%), whilst the largest group of the sample was that of the 46-90 years old with a total of 128 patients (69.2%). The patients who experienced at least one feature of dental anxiety established via the DAS (moderate, high and severe) made up a total of 111 patients from the sample (60%) in contrast to those for whom the DAS did not detect any level of anxiety (40%). Similarly, the patients who required analgesia made up a total of 103 (55.7% of the total sample) dividing the analgesic options quite equally among themselves (paracetamol for 27.6% of the sample versus ibuprofen for 28.1% of the sample). Regarding the anaesthesia administered: 46 men were given articaine and the other half of the men were given lidocaine. The same was done with the women regarding anaesthesia, resulting in 49.7% of the total sample being anaesthetised with articaine and 50.3% with lidocaine (Table 1).

**Table 1:** Data collected regarding the study population.

Qualitative variables	Categories	Frequency	Percentage (%)
Sex	Male	92	49.7
	Female	93	50.3
Age groups	18-45 years old	57	30.8
	45-90 years old	128	69.2
Type of anaesthesia	Articaine	92	49.7
	Lidocaine	93	50.3
Need for analgesia	Yes	103	55.7
	No	82	44.3
Type of medication	No medication	82	44.3
	Paracetamol 650 mg	51	27.6
	Ibuprofen 400 mg	52	28.1
Level of anxiety (DAS)	Low-No anxiety (1)	74	40.0
	Moderate (2)	38	20.5
	High (3)	36	19.5
	Severe (4)	37	20.0

On the other hand, regarding the quantitative variables of the study (Table 2), the average age of the study population was 55.9, and the average number of carpules used in the study was 1.6 carpules. Regarding the average of the pre-operative physiological variables, we have the following figures: systolic BP (144.9 mmHg), diastolic BP (76.4 mmHg) and HR (75.1 beats per minute - bpm), in comparison to the post-operative variables which were: systolic BP (145.4 mmHg), diastolic BP (77.7 mmHg) y HR (75.3 beats per minute - bpm).

**Table 2:** Data collected regarding the physiological variables in the study.

Quantitative Variables	Minimum	Maximum	Average ± Standard Deviation
Age	18 years	90 years	55.9 ± 18.1 years
Carpules used	1 unit	5 units	1.5 ± 0.7 units
Pre-operative systolic BP	100 mmHg	210 mmHg	144.9 ± 24.4 mmHg
Post-operative systolic BP	97 mmHg	209 mmHg	145.4 ± 23.4 mmHg
Pre-operative diastolic BP	52 mmHg	112 mmHg	76.4 ± 10.4 mmHg
Post-operative diastolic BP	55 mmHg	115 mmHg	77.7 ± 10.9 mmHg
Pre-operative HR	53 bpm	161 bpm	75.1 ± 12.3 bpm
Post-operative HR	45 bpm	114 bpm	75.3 ± 10.2 bpm
Duration of extraction	3 seconds	977 seconds	121.2 ± 156.3 seconds
Days during which analgesia was required	0 days	4 days	0.9 ± 1.0 days

### Pre-operative and post-operative physiological parameters

Spearman’s rho coefficient shows that all of these are connected, the strongest relationships being those between the same variables (pre-operative and post-operative systolic BP, pre-operative and post-operative diastolic BP and pre-operative and post-operative HR) in the same patient before and after the intervention. The correlation coefficients are 0.913 for pre-operative and post-operative systolic BP, 0.866 for pre-operative and post-operative diastolic BP and 0.865 for pre-operative and post-operative HP. This indicates that there is a clear relationship between the physiological variables before the extraction and after.

What was studied next was whether or not there were significant differences in each of the physiological parameters before and after the operation. This is determined by using the Student’s t-test for paired samples, as data has been found to be normal (p-value < 0.05, Kolmogorov-Smirnov test) and homoscedastic (*P*-value < 0.05, Levene test). Table 3 shows that statistically significant differences only exist in diastolic BP (*P*-value=0.001). These changes in diastolic BP, which measures the residual force exerted by the heart on the artery walls between each heartbeat, represent a variance in the patient’s physiological state that could be due to the state of anxiety they find themselves in when sitting in the dentist’s chair at the beginning of the intervention as opposed to at the end of it.

**Table 3:** Significance level of the averages of pre-operative and post-operative physiological variables.

	Average	Standard deviation	*P* value
Pre-operative systolic BP – Post-operative systolic BP	−0.519	10.885	0.518
Pre-operative diastolic BP – Post-operative diastolic BP	−1.362	5.421	*0.001*
Pre-operative HR – Post-operative HR	−0.189	8.058	0.75

### Relationship between the physiological parameters and the DAS

Cohen’s f was used to study the relationship between level of anxiety and the physiological variables, with each indicating a high effect, with numbers higher than 0.8.

**Figure 1:**
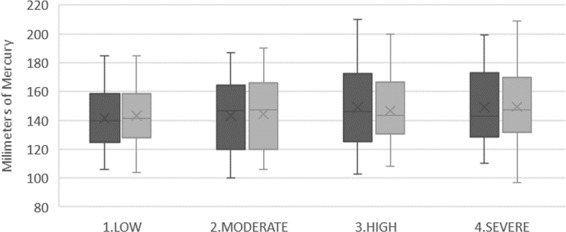
Relationship between pre-operative and post-operative systolic blood pressure in regards to the DAS (n = 185). Dark colour represents pre-operative measures and light colour represents post-operative measures to each degree of anxiety.

**Figure 2:**
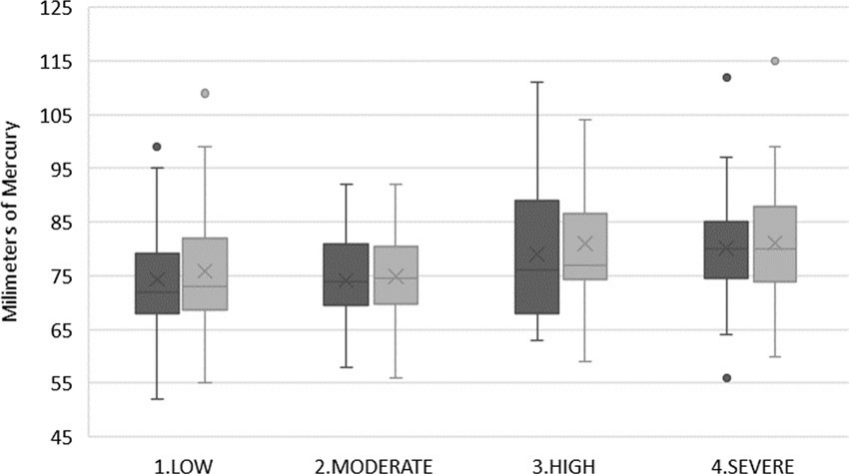
Relationship between pre-operative and post-operative diastolic blood pressure in regards to the DAS (n = 185). Dark colour represents pre-operative measures and light colour represents post-operative measures to each degree of anxiety.

**Figure 3:**
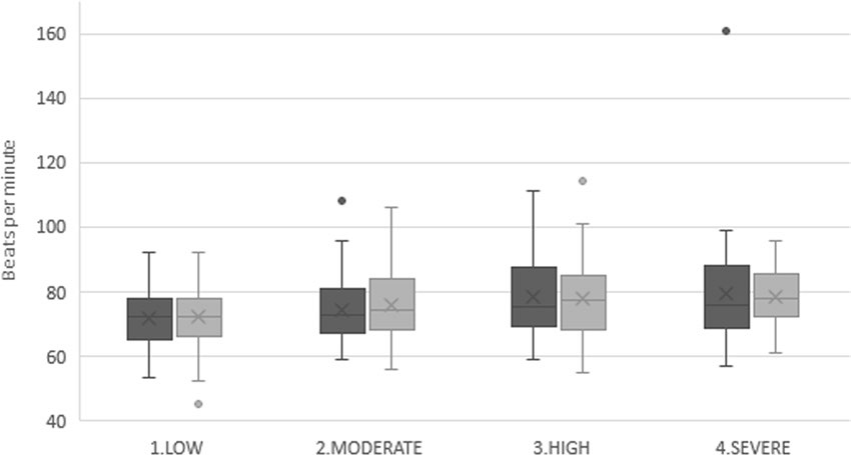
Relationship between pre-operative and post-operative heart rate in regards to the DAS (n = 185). Dark colour represents pre-operative measures and light colour represents post-operative measures to each degree of anxiety.

The significance level was observed between the averages of systolic and diastolic BP and HR before and after the extraction in relation to the levels of anxiety obtained in the DAS. The Kruskal-Wallis non-parametric test was used to analyse the significance level. The results are shown in Figs. 1–3, with statistically significant differences found in the relationship between the value obtained in the DAS and pre-operative diastolic BP (*P* = 0.011) and post-operative diastolic BP (*P* = 0.004), as well as the pre-operative HR (*P* = 0.027) and post-operative HR (*P* = 0.013). However, no statistically significant values were found for pre-operative systolic BP (*P* = 0.613), or post-operative systolic BP (*P* = 0.810) in regards to the different levels of anxiety.

The *P*-value of the previous systolic BP averages for the four different levels of anxiety is the same (0.613). On the other hand, the *P*-value of the post-operative systolic BP averages is 0.810 for the four levels of anxiety. Therefore, these results are not statistically significant and suggest there is no relationship between the systolic BP studied and the results obtained via the DAS (Fig. 1).

Regarding the previous diastolic BP averages for the four levels of anxiety, *P*-value was 0.011. On the other hand, the *P*-value of the post-operative diastolic BP averages was 0.004. These results are therefore statistically significant and indicate that a relationship exists between the diastolic BP studied and the values obtained via the DAS (Fig. 2).

Finally, the *P*-value for the previous HR averages for the four different levels of anxiety was found to be 0.027. On the other hand, the post-operative HR average for the same four different levels of anxiety was 0.013. Therefore, the results turned out to be statistically significant and explain the relationship between the HR and DAS (Fig. 3).

### The relationship between level of anxiety, anaesthesia used and the need for post-operative analgesia

To evaluate the relationship between level of anxiety and analgesia, Pearson’s chi-squared test was used, as these constituted two categorical, or qualitative, variables.

A *P*-value was obtained, which indicated that the two variables are connected (Table 4). Furthermore, in Table 4, the patients found in each category are shown. For example, out of those with anxiety level 1 (low), the majority (40/74) did not take medication, or took paracetamol (24/74). For anxiety levels 2 and 4, no significant differences were observed regarding frequencies. However, the lowest frequency of patients who took paracetamol is observed for anxiety level 3, (6/36). These results indicate that patients with low levels of anxiety (1) tend to not take medication, or, if analgesia is needed, prefer to take paracetamol 650 mg.

**Table 4:** Relationship between level of anxiety and the medication administered post-treatment (% of patients). Pearson’s chi-squared test.

		Grade of Anxiety (DAS)				*P* value
		1	2	3	4	0.038
**Medication**	No medication	21.7 (*)	8.2	7.6	7.1	
	Paracetamol 650 mg	13 (*)	5.4	3.3	5.4	
	Ibuprofen 400 mg	5.4	6.5	8.7 (**)	7.6 (**)	

^*^Comparison between levels of anxiety and medication. Patients with Level 1 anxiety (low) tend to not take medication or medicate themselves with paracetamol 650 mg.

**Comparison between levels of anxiety and medication. Patients with Level 3 and 4 anxiety (high and severe) tend to take ibuprofen 400 mg.

When the Z (normal distribution) test was carried out for the differences in proportions (see Table 4 where the corresponding number is indicated in parenthesis), it was possible to observe that differences are indeed significant for the intake of ibuprofen, among anxiety levels 1, 3 and 4. This result leads us to the conclusion that patients who show higher levels of anxiety (high and severe) tend to select ibuprofen 400 mg as their first choice of medicine to prevent a possible future pain which might never appear after a simple extraction.

On the other hand, to study the possible relationship between the anaesthesia used in the study (articaine and lidocaine) and the need for analgesia after the intervention, Pearson’s chi-squared test was carried out and a *P*-value = 0.948 was obtained, which indicates that the two variables are not connected.

### Other parameters studied regarding the extraction time

Other parameters that ultimately did not influence the final results of our study are the periodontal state of the tooth, the need for prior antibiotic intake and the extraction time with respect to the degree of anxiety.

To establish the statistical correlation regarding the four possible degrees of periodontal involvement of a tooth, the Kruskal-Wallis test was performed for independent samples in which a significance of *P* = 0.0001 < 0.05 was obtained (Table 5). This reflects, as expected, that the highest degree of periodontal affectation directly intervenes in the reduction of extraction time and vice versa.

**Table 5:** Relationship between the periodontal state, antibiotics prior to extraction and DAS related to the extraction time.

Variables	Categories	Media (extraction time in seconds)	*P* value
Periodontal state	0	139.79 ± 170.64	*0.0001*
	I	100.8 ± 108.26	
	II	53.4 ± 82.44	
	III	16.4 ± 12.91	
Antibiotic previous extraction	Yes	114.3 ± 146.3	0.772
	No	126.1 ± 163.8	
Grade of anxiety (DAS)	No anxiety	54.4 ± 80.2	0.393
	Moderate	119 ± 167.7	
	High	183.3 ± 217.9	
	Severe	125.7 ± 176	

Similarly, to assess the need for prior antibiotic intake or not and how it affects the extraction time, the Mann-Whitney U test was used for independent samples with a significance of *P* = 0.77 > 0.05 (Table 5). Therefore, a non-statistically significant result was obtained that reinforces the idea that the extraction time is equal in patients who require drug-antibiotic treatment prior to extraction and those who do not need it.

Finally, to assess how the extraction time was affected by the different degrees of anxiety obtained, the Kruskal-Wallis test was used for the independent samples, a significance of *P* = 0.393 > 0.05 was obtained (Table 5). Thus, no statistically significant differences are obtained between the different anxiety groups regarding the extraction time.

## Discussion

Dental extractions are a common procedure in the dental clinic, with a short recovery time if no problems arise, such as the occurrence of alveolitis. However, psychological factors mean that this could be a deeply stressful situation for the patient.

The correct management and determination of the patient we will be treating is fundamental, as shown in the study published in 2012 by Hierons *et al*. In this paper, patients took the MDAS test in order to establish if it is better to use conscious sedation or just local anaesthetic in an extraction^20^. However, conscious sedation requires equipment and highly qualified and specific human resources which not all clinics have access to.

The aim of our study was to identify which patients have a higher level of anxiety by using Corah’s Anxiety test in relation to various physiological variables, such as pre and post-operative BP and pre and post-operative HR, and how these affect possible post-treatment analgesia. No significant differences between sexes were found in relation to the different variables studied, unlike in a study by Gadve *et al*.^21^. in which there were higher significant differences in women in comparison to men in both systolic and diastolic BP. However, our study does not show any significant differences between sexes regarding HR, which coincides with what was published by Gadve *et al*.

In 1987, Meyer proposed that emotional stress masked physiological changes in blood pressure and heart rate which could be caused by local anaesthetic with a vasoconstrictor^22^. In fact, in our study we saw no evidence of difference between the two local anaesthetics used (both lidocaine 2% and articaine 4% were used with epinephrine 1:100.000) and the physiological variations of BP and HR. This can support the idea that it is in fact the level of anxiety experienced by a patient which can be a determining factor in pre and post-operative variables. Therefore, the fear and anxiety intrinsic to the intervention exist in the patient, as demonstrated by Klepac *et al*.^13^ in 1980. After electrically stimulating the patient with different tests, it was found that the patients with more fear responded with greater anxiety to non-dental stimulation, and with higher levels of anxiety to dental stimulation. This could lead to the variation of physiological parameters related to anxiety that we have measured.

In our study, statistically significant differences were seen between pre and post-operative diastolic BP. Nonetheless, in contrast to our study, Liau *et al*.^23^ found no differences in the physiological parameters that they measured after anaesthetising the patient. They took three readings of blood pressure and heart rate at five, ten and fifteen minutes after anaesthetising the patient and did not find any statistically significant differences in any of the variables.

The pre and post-operative diastolic variable in our study did appear to be statistically significant, though this could be due to the fact that in our study the post-operative measurement was taken after the extraction was finished and thus, later than in Liau *et al*.^23^, which means that this time difference regarding taking blood pressure could be responsible for these results.

Therefore, it is confirmed that there are changes in the physiological variables in the dental clinic before and after treatment and that these are not due to elements intrinsic to the dental intervention, such as the use of vasoconstrictors associated to local anaesthetic, as was already demonstrated by Silvestre *et al*. in 2001^24^, but, in fact, probably due to the fear and anxiety that a patient might experience. This level of nervousness in patients is caused by the negative psychological perception of the treatment to be carried out, more than the difficulty involved or the actual surgical task, as claimed by Raocharernporn *et al*.^15^. In this study, the patients were not administered a test measuring the level of anxiety they might manifest, as it was assumed that fear could be directly evaluated as a variation of BP or HR.

In our study we did, in fact, observe statistically significant differences for the different levels of anxiety obtained via Corah’s Test and pre and post-operative diastolic BP, as well as pre and post-operative HR. The results indicate that the most significant differences are found, above all, for moderate and severe levels of anxiety, similarly to what is described in a study by Sharma *et al*., where it was observed that, after anaesthetising the patient, BP and HR values rose in Corah’s three levels of anxiety (medium, high and severe)^25^. The physiological changes evaluated in regards to the levels of anxiety obtained via Corah’s Test were also seen in Liau *et al*.’s study, which rose in the three groups, especially in the moderate and severe groups^23^.

Regarding the possible differences obtained between sexes, in our study no statistically significant differences were seen between the groups of men and women and the values obtained with Corah’s Test. Despite not being significant, there was a higher percentage of severe anxiety in the group of women (27%) with respect to the group of men (13%). This data is similar to that published by Dobros *et al*. in their study, where higher percentages of anxiety appear in women than in men, but without any statistical significance^11^. Tarazona-Alvarez *et al*. did find significant differences between men and women in wisdom tooth extraction, where women obtained higher levels of anxiety when completing the DAS^26^. In contrast, Liau *et al*. found that the group of men presented with a higher percentage of severe anxiety. However, the authors highlight that the values obtained could be due to the particular conditions of their study^23^.

In our study it was found that, even though there were no statistically significant differences between the DAS and the variable of sex, there were significant differences between the values obtained via the DAS and the variable of “post-operative analgesia”. Patients with higher ranges of anxiety took higher quantities of pharmaceuticals compared to patients with low or medium anxiety. These results coincide with those published by Kazancioglu *et al*. in 2017, where it is shown that patients with high levels of anxiety have a greater tendency to take analgesic medication, especially after treatments of a surgical nature^27^. In fact, other researchers, such as Torres-Lagares *et al*., though using different scales to measure dental anxiety such as the “Spielberger State-Trait Anxiety Inventory-Trait and State” (STAI-S) for the analysis of medication taken after the intervention, found that the patients who scored higher in the anxiety scale were the same who later took higher quantities of pain medication, in this case ibuprofen 600 mg^28^. These results show a clear consistency with those obtained by Wang *et al*. in 2017^29^, where it was found that high levels of anxiety (obtained via a different scale to the DAS), normally due to previous bad experiences, result in a higher sensation of post-operative pain, which leads to a greater intake of analgesic medication. This not only occurs with post-operative pain, but even in endodontic treatments, patients with higher levels of anxiety tend to have higher expectations of the intensity of intraoperative pain that they may suffer^14^.

With regards to the intake of anti-inflammatory analgesics, we can see in studies published by Beaudette *et al*. how, after dental implant surgery or periodontal surgery, these greatly depend on how much pain the patient expects to experience^30^. This can be linked to the results obtained in our study, where patients with moderate or severe anxiety levels tended to take more ibuprofen 400 mg than those with low levels of anxiety. Beaudette’s results also indicate that the peak of maximum pain occurs on the same day of extraction and the day after^30^. These results coincide with ours, where we have seen that out of all the patients who expressed pain, 89.2% placed it within the first 24 hours after the dental extraction.

Other studies, such as Deshpande *et al*. found similar parameters as in our studies in regards to analgesia and how long it was used for^31^, in that no significant differences were found between pain control with ibuprofen or paracetamol and that the moment of highest use of analgesia in both groups was situated within the first 24 hours after the extraction. On the other hand, Al-Khateeb and Alnahar^32^, despite finding similar results in their study regarding the peak of maximum need for analgesia in the same day of extraction, conclude by recommending that dentists offer analgesia during at least a week after the extraction. This recommendation goes against the results obtained in our study, in which the majority of patients tended to take analgesics on the day of the extraction or at most 24 hours after it. Therefore, there is no evidence of any need to maintain analgesia for a week when there is an absence of objective pain.

However, despite not measuring anxiety levels, Al-Khateeb and Alnahar’s 2008 study indicates that the patients who perceive the most fear in regards to an anaesthetic injection are those who are more likely to take post-operative analgesia^32^. These results do coincide with those shown by our study, where patients with higher levels of anxiety (high and severe) tended to take greater quantities of medication, specifically ibuprofen 400 mg, while patients with lower levels of anxiety (no or low anxiety) have a greater tendency to not use analgesia, or if they do, they are more inclined to use paracetamol 650 mg.

We have seen differences in pre-operative and post-operative diastolic BP with a clear correspondence to the data obtained in the classic DAS. We have also seen that patients with higher levels of anxiety have a greater tendency to take anti-inflammatory medication compared to patients with low anxiety, who only require analgesia or no medication. In this way, our study hypothesis is verified. These results lead us to believe that extraction begins before the intervention in the dental clinic. If we are able to reduce anxiety levels in the patient, the intake of medication after the intervention could also be reduced, either reducing or eliminating the consumption of anti-inflammatory analgesics with all the benefits that this would have on the patient’s general health.

## Data Availability

The datasets generated during the current study are available from the corresponding author on reasonable request.
